# Adaptive Changes in Endurance Athletes: A Review of Molecular, Echocardiographic and Electrocardiographic Findings

**DOI:** 10.3390/ijms26178329

**Published:** 2025-08-28

**Authors:** Michał Janik, Dominika Blachut, Łukasz Czogalik, Andrzej Robert Tomasik, Celina Wojciechowska, Tomasz Kukulski

**Affiliations:** 2nd Department of Cardiology, Medical University of Silesia in Katowice, 41-800 Zabrze, Polandlukczog@gmail.com (Ł.C.); atomasik@sum.edu.pl (A.R.T.); cwojciechowska@sum.edu.pl (C.W.); tkukulski@sum.edu.pl (T.K.)

**Keywords:** athlete’s heart, biomarkers, myocardial regeneration, echocardiography, exercise, marathon runners, molecular mechanisms of adaptation, running

## Abstract

Regular physical activity has a beneficial impact on the cardiovascular system. However, the intense and prolonged exertion typical of professional athletes and amateur marathon runners can lead to adaptive changes in the heart. These changes encompass both structural and functional modifications, which may have positive or negative effects on cardiac function and contribute to the development of so-called “athlete’s heart.” Prolonged exercise induces adaptations at the molecular and cellular levels, including altered gene expression and remodeling of myocardial proteins. It may also cause transient elevations in biomarkers such as N-terminal pro-brain natriuretic peptide (NT-proBNP) and high-sensitivity troponin. Some athletes experience cardiac arrhythmias, including atrial fibrillation. Morphological changes, such as myocardial hypertrophy or chamber dilation, can be assessed using echocardiography. Studies have reported potentially benign valvular abnormalities, as well as cases of myocardial fibrosis and arrhythmias. Early diagnosis of cardiac conditions in marathon runners is essential for effective prevention and health monitoring. This article reviews the current data on cardiac changes in endurance athletes, based on the literature from the past decade.

## 1. Introduction

Regular physical activity offers numerous health benefits, including a positive impact on the cardiovascular system. However, the intense and prolonged exercise characteristic of professional athletes and amateur marathon runners or triathletes can lead to specific load-related adaptive changes in the heart. These changes involve both structural and functional modifications of the myocardium, which may have either beneficial or adverse effects on cardiac function, sometimes resulting in the development of so-called “athlete’s heart” [1,2,3,4]. Intense and sustained physical exertion induces not only macrostructural alterations in the heart but also adaptations at the molecular and cellular levels. In response to increased hemodynamic stress, changes in gene expression, activation of signaling pathways, and remodeling of myocardial proteins occur [5].

The changes can be classified as either reversible or irreversible. From a cardiologist’s perspective, early detection of adverse irreversible changes is crucial. The current guidelines of the European Society of Cardiology (ESC) on sports cardiology and exercise in individuals with cardiovascular disease recommend that a cardiological evaluation, conducted prior to initiating or continuing intensive training, should include a detailed medical history, physical examination, and a 12-lead resting electrocardiogram (ECG) to detect potentially dangerous arrhythmias or signs of myocardial hypertrophy. In equivocal cases or in individuals at higher risk (e.g., those with a positive family history, cardiac symptoms, or ECG abnormalities, further diagnostic evaluation is recommended. This includes transthoracic echocardiography, exercise testing, and 24 h Holter ECG monitoring. In selected athletes, particularly those involved in high-intensity endurance sports, advanced imaging techniques such as cardiac magnetic resonance (CMR) may be indicated to assess for myocardial fibrosis, benign structural changes, or to evaluate right ventricular function. The ESC guidelines also emphasize the importance of interpreting diagnostic findings within the context of physiological adaptations to training—referred to as “athlete’s heart”—which may mimic pathological changes, but are typically reversible and not associated with an increased risk of sudden cardiac death [6].

Intense exercise can cause a transient increase in biomarkers commonly used in clinical practice to assess cardiac function, such as N-terminal pro-brain natriuretic peptide (NT-proBNP) and high-sensitivity troponin [7,8]. These changes are more likely to reflect the body’s adaptation to significant and prolonged physical exertion rather than permanent cardiac damage.

Although such changes are usually physiological and reversible, intense physical activity can lead to cardiac arrhythmias, such as atrial fibrillation (AF) or ventricular extrasystoles, particularly in older individuals who train intensively [9,10]. This type of physical stress also induces morphological remodeling of the heart, which can be assessed using echocardiography or cardiac CMR. Observed changes include myocardial hypertrophy, dilatation of cardiac chambers, and alterations in ejection fraction [11,12].

Studies indicate that endurance athletes may also develop valvular abnormalities. Physical activity appears to promote mild mitral and tricuspid regurgitation. In addition, athletes with pre-existing valvular conditions—such as bicuspid aortic valve—are also subject to evaluation [13]. Cases of right ventricular dysfunction in otherwise “healthy” athletes have been described in the literature. Other pathologies observed in asymptomatic endurance athletes include myocardial fibrosis, coronary artery atherosclerosis, and arrhythmias [14].

With the growing popularity of endurance sports, accurate assessment of the effects of intense physical activity on the heart is becoming increasingly important. This article reviews recent findings on cardiac adaptations to strenuous exercise, with particular emphasis on structural, functional, electrophysiological, and molecular changes observed in endurance athletes, including marathon runners.

While current physical activity guidelines highlight substantial health benefits from 150 min of moderate or 75 min of vigorous exercise per week, endurance athletes typically train 15 to 20 times beyond these recommendations [15,16]. Both the American Heart Association (AHA) and the ESC recommend cardiovascular screening before participation in competitive sports to identify high-risk individuals [17,18]. Studies also show that regular training and completing a first marathon at a slower pace may help reduce central blood pressure and arterial stiffness [14].

Early detection of cardiac problems in marathon runners is crucial for effective prevention and health monitoring. There is an urgent need to better understand the mechanisms underlying the changes observed in the athlete’s heart. Although marathon running is becoming increasingly popular—particularly among amateur athletes—data on the effects of this form of exercise on the heart remain limited. Despite the growing number of studies, there are still no clear guidelines for monitoring the cardiovascular health of marathon runners. This article presents recent findings on the molecular mechanisms as well as echocardiographic and ECG changes observed in endurance athletes. To prepare this manuscript, a comprehensive review of scientific databases—including ClinicalKey, PubMed, and EMBASE—was conducted, with a focus on publications from the last decade (2015–2025).

## 2. Impact of Exercise on Cardiac Pathogenesis

In endurance sports such as marathon running, the structural dimensions of the athlete’s heart may resemble those observed in dilated cardiomyopathy and arrhythmogenic right ventricular cardiomyopathy, and in some cases may even mimic hypertrophic cardiomyopathy [19,20,21,22]. Intense physical exertion can lead to adverse changes in cardiac function, such as accelerated development of coronary atherosclerosis, myocardial fibrosis, and right ventricular dysfunction [5,23].

After intense physical exertion, professional athletes may experience transient impairment of left ventricular function. Growing evidence suggests that due to differing compensatory mechanisms in the pulmonary and systemic circulation, exercise-induced cardiac overload affects and manifests in the right ventricle (RV) more prominently and earlier than in the left ventricle (LV) [24,25,26,27].

**Figure 1:**
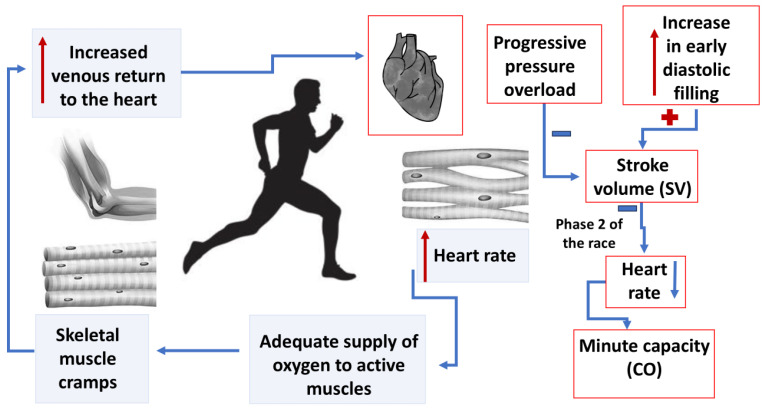
Hemodynamic changes in the right heart during exercise: a schematic diagram.

Systematic, intense training leads to moderate thickening of the left ventricular muscle and enlargement of the chamber, while maintaining normal systolic and diastolic function—a phenomenon known as the “athlete’s heart” [21,22]. This complex adaptation is a form of cardiac remodeling. Skeletal muscle contractions during intense running increase venous return to the heart. During running, stroke volume (SV) increases due to enhanced ventricular filling in early diastole, although it may decrease in response to increasing pressure overload [28]. Simultaneously, during the middle phase of the run, heart rate (HR) increases to ensure adequate oxygen delivery to the working muscles. In the later part of the race, increased SV allows for a reduction in HR, thus adjusting cardiac output (CO) accordingly [29]. Changes in HR and SV together contribute to the overall increase in CO during exercise. Cardiac workload remains submaximal and relatively stable for most of the run duration [27,30,31,32]. This hemodynamic pattern is illustrated in Figure 1. The reduced resting heart rate commonly observed in endurance athletes is most likely due to long-term, intense endurance training, which leads to enhanced vagal tone and reduced sympathetic activity in marathon runners [33].

Endurance effort significantly increases myocardial oxygen demand and SV. In the early phase of exertion, there is a rapid increase in HR due to the withdrawal of tonic vagal influence and activation of the sympathetic nervous system. This results in the release of adrenaline and noradrenaline into the circulation. These catecholamines increase myocardial contractility and heart rate, leading to elevated SV. During prolonged endurance activity, features of transient cardiac dysfunction may emerge, with an average drop in left ventricular ejection fraction (LVEF) of up to 2% after endurance exercise. This is observed in untrained individuals as well as ultra-endurance athletes [34,35,36].

Repeated contractions of muscle fibers during intense physical exertion induce both mechanical and metabolic stress, associated with mitochondrial dysfunction and a shift in the adenosine diphosphate to adenosine triphosphate ratio. This leads to damage of the sarcolemma and extracellular matrix, mitochondrial swelling, dilation of T-tubules, and fragmentation of the sarcoplasmic reticulum, resulting in increased cell membrane permeability and release of muscle proteins into the circulation. Additionally, disturbances in calcium homeostasis, oxidative stress, and inflammation contribute to the progression of muscle injury. In response to this condition, increased levels of inflammatory mediators such as interleukins (IL)—IL-1β, IL-6, IL-8, IL-1ra, and IL-10—are observed, which are characteristic of endurance-exercise-induced muscle damage [37,38].

### 2.1. Molecular Changes and Biomarkers

Studies indicate that intense physical exertion, particularly during long-distance running, can acutely stress the cardiovascular system. Elevated levels of biomarkers of myocardial injury and hemodynamic dysfunction are commonly observed. Increased concentrations of markers such as cardiac troponins, creatine kinase-MB fraction (CK-MB), NT-proBNP, and mid-regional pro-adrenomedullin (MR-proADM) suggest transient or chronic cardiac stress, and even cardiomyocyte injury [39,40].

To assess myocardial microdamage after intense exertion, biomarkers of cell necrosis—such as troponin I (TnI) and creatine kinase (CK)—have been used, as well as markers of cardiac overload, such as NT-proBNP and MR-proADM, and indicators of endogenous stress such as copeptin [38,39,40,41]. Other markers have also been identified, such as copeptin (a marker of endogenous stress) and pro-inflammatory cytokines like IL-6 and tumor necrosis factor (TNF-α), which increase after intense physical exertion and may play a role in the pathogenesis of myocarditis, cardiac arrhythmias, and fibrosis [41,42,43,44,45].

The underlying mechanisms of these changes include necrosis of both myocardial and skeletal muscle cells as well as cardiac overload, which may ultimately lead to permanent structural changes in the cardiovascular system [27]. In endurance sports, elevated concentrations of myocardial injury biomarkers—such as NT-proBNP and troponin—and signs of cardiac dysfunction are commonly observed. These changes may contribute to subclinical myocarditis and cardiac remodeling, increasing the risk of arrhythmias. The concentrations of cardiac injury markers—including cardiac troponin, CK-MB, and NT-proBNP—significantly increase both during and after marathon running [46]. CK-MB, a classical marker of myocardial injury, may be elevated in up to 8% of athletes, particularly affecting the RV [47].

After running, a clear increase in blood levels of troponin T (cTnT)—a protein primarily found in the thin myofilaments of myocardial fibers—is observed, suggesting reversible myocardial injury and transient cardiac dysfunction. In amateur marathon runners, an increase in cTnT levels and transient myocardial injury occurs more frequently than in professionals [46,48]. Intense physical exertion may lead to a temporary increase in cardiomyocyte sarcolemma permeability, due to mechanical stress, excessive reactive oxygen species (ROS) production, acid–base imbalance, and passive transport of cardiac troponins from the intracellular space to the extracellular compartment. As a result of stretching and mechanical load, the cell membrane may undergo transient damage, leading to cTn release into circulation. This phenomenon is especially pronounced during prolonged and high-intensity exertion [49].

Although the elevation in troponin levels is often transient and reversible, it can be misleading, as it may suggest an acute coronary syndrome in asymptomatic individuals. However, in athletes, increased troponin levels most often result from reversible increases in cardiomyocyte membrane permeability rather than necrosis [50]. Oxidative stress and electrolyte disturbances associated with intense endurance exercise may increase membrane permeability, elevating troponin concentrations in the blood [51].

Studies show that elevated NT-proBNP levels correlate with the duration and intensity of exercise, as well as with volume and pressure overload parameters. This may indicate ventricular overload—especially of the RV—and a neurohormonal adaptive response of the heart. NT-proBNP may increase 5- to 10-fold after exercise in those participating in endurance events [48,52].

High-intensity physical exertion selectively activates the NF-κB pathway, a protein complex regulated by TNF-α and p38 MAPK, which belongs to the class of stress-sensitive kinases. These pathways are involved in immune responses, cell cycle control, and the regulation of various cellular processes. Their activation plays an important role in myocardial remodeling by promoting fibrosis, which may represent a mechanism leading to AF in endurance athletes [53].

Endurance athletes demonstrate elevated levels of plasma markers of collagen synthesis and degradation, including tissue inhibitor of matrix metalloproteinases-1 (TIMP-1), carboxy-terminal telopeptide of type I collagen (CITP), carboxy-terminal propeptide of type I procollagen (PICP), galectin-3 (Gal-3), and circulating profibrotic microRNA-21. The highest TIMP-1 concentrations correlate with left ventricular hypertrophy. A significant increase in plasma levels of soluble vascular cell adhesion molecule-1 (sVCAM-1) is also observed. This molecule plays a key role in inflammatory cell adhesion and leukocyte migration through the vascular endothelium. A study conducted in white runners participating in high-intensity training found that sVCAM-1 may serve as a useful biomarker for assessing and monitoring adverse structural and functional changes in the left atrium (LA), with levels positively correlating with LA volume [34,47,52,54].

Gal-3 is a marker of myocardial remodeling and fibrosis, released in response to mechanical stretching of cardiomyocytes and inflammatory processes induced by activated macrophages. Our study showed that physical exertion leads to a transient increase in Gal-3 levels in amateur athletes. Notably, a greater post-race increase in Gal-3 concentration was observed in participants with lower LVEF and reduced RV contractility, suggesting that exercise-induced cardiac fibrosis may lead to transient left ventricular dysfunction. Moreover, myocardial ischemia may initiate an inflammatory response that promotes macrophage infiltration and consequently contributes to the development of myocardial fibrosis [55,56,57,58].

The study observed leukocytosis, neutrophilia, and elevated levels of muscle damage markers, pro- and anti-inflammatory cytokines (IL-6, IL-8, IL-10, TNF-α, MIP-1), as well as myokines and growth factors such as decorin, growth differentiation factor 15 (GDF15), brain neurotrophic factor (BDNF), follistatin, and fibroblast growth factor 21 (FGF-21) directly after the race. Simultaneously, decreased levels of myostatin, musclin, IL-15, and apelin were noted, which persisted up to 72 h post-exercise. Plasma lactate dehydrogenase activity correlated with levels of inflammatory cytokines (IL-10 and TNF-α) but showed no relationship with the myokine response [59].

Endurance exercise triggers an initial pro-inflammatory response in which neutrophils and pro-inflammatory M1 macrophages play a key role. This is followed by a compensatory anti-inflammatory response mediated by M2 macrophages and T cells, including regulatory T lymphocytes (Tregs) and CD8+ cells. This process leads to a temporary state of immunosuppression [37,60].

Studies have shown that IL-6 stimulates myogenic differentiation in both C2C12 myoblast cell lines and primary human myoblasts. It also promotes protein synthesis in C2C12 myotubes and the proliferation of murine myoblasts and human satellite cells. Animal model studies have highlighted the role of IL-6 in activating M2 macrophages, which promote angiogenesis and tissue regeneration processes, as well as enhance cell proliferation [61,62,63].

Follistatin exhibits myogenic properties resulting from its direct inhibition of myostatin binding to the activin type IIb receptor and suppression of Smad3 protein phosphorylation. This leads to increased protein synthesis via the mTOR/S6K/S6RP signaling cascade, supporting skeletal muscle mass development. Follistatin may correlate with BDNF levels, a factor essential for activation and proliferation of muscle satellite cells after injury. Literature reviews indicate that BDNF acts on the myocardium by inhibiting cardiomyocyte apoptosis and mitochondrial dysfunction, supporting angiogenesis, enhancing cardiomyocyte contractility, and regulating calcium homeostasis through tropomyosin receptor kinase B (TrkB) signaling pathways [64,65,66,67].

Mitochondrial and endoplasmic reticulum dysfunction induce FGF-21 expression in skeletal muscles. FGF-21 regulates PI3K-AKT signaling, activates activating transcription factor 4 (ATF4) in muscles, and participates in the elimination of damaged mitochondria through mitophagy, as well as in muscle fiber type switching. In this way, it affects both muscle mass and function. Additionally, FGF-21 demonstrates similar metabolic properties to IL-6 in skeletal muscles. A negative correlation was observed between the FGF-21 response and levels of decorin and apelin, suggesting that stronger FGF-21 activation may counteract the post-exercise decline of these proteins [66,68,69].

Myostatin binds to activin type I and II receptors, leading to phosphorylation and activation of SMAD family proteins. The SMAD-2/3 complex binds to SMAD-4, initiating the transcription of genes responsible for protein catabolism. Additionally, myostatin is involved in protein degradation via the ubiquitin–proteasome system and autophagy. Exercise-induced inflammatory mediators such as IL-10 and IL-8 showed correlations with changes in musclin levels—a protein with a region homologous to the natriuretic peptide family. Musclin likely supports skeletal muscle oxidative capacity by stimulating mitochondrial biogenesis [66,70,71,72,73].

Suppression of tumorigenicity 2 (ST2) belongs to IL-1 receptor family and functions as a receptor for the IL-33 cytokine. IL-33 is secreted by cardiac fibroblasts in response to cell injury and acts by binding to the membrane-bound form of the ST2 receptor (ST2L) on cardiomyocytes. The IL-33/ST2 axis is activated in the heart in response to mechanical overload or injury, leading to the inhibition of myocardial fibrosis and hypertrophy. Marathon running causes a significant increase in sST2 concentration, with higher levels observed in runners with better performance. It has been shown that male sex, exercise intensity, and greater body weight loss during the marathon are directly associated with sST2 levels [74,75].

Beyond biomarker changes, recent studies have elucidated key molecular signaling pathways that mediate exercise-induced cardiac remodeling. Physiological hypertrophy is largely driven by IGF-1/PI3K–Akt–mTOR activation, whereas pathological remodeling involves Ang II and endothelin-1 signaling through MAPK and calcineurin/NFAT pathways, promoting fibrosis and apoptosis. In parallel, activation of TGF-β/SMAD signaling contributes to extracellular matrix expansion, consistent with elevations in galectin-3, TIMP-1, and PICP observed in endurance athletes. Oxidative stress and mitochondrial dysfunction further exacerbate cellular injury, while NF-κB and inflammasome activation drive cytokine release, linking inflammation to structural remodeling. These pathways not only explain the biomarker profiles documented in athletes but also provide mechanistic insight into the development of fibrosis, diastolic dysfunction, and arrhythmogenic substrates [76,77,78,79,80,81,82]. A summary is presented in Table 1.

### 2.2. Cardiac Pathological and Physiological Alterations

#### 2.2.1. Cardiac Fibrosis

Long-term stress and chronic volume overload resulting from intensive training can lead to progressive remodeling of myocardial fibers in the subendocardial and subepicardial layers of the heart muscle. In athletes, post-exercise myocardial fibrosis has been observed—ranging from minor changes in the RV, which may represent a benign effect of chronic activity, to extensive areas of intramyocardial and diffuse fibrosis [46,47,48,49,50]. Myocardial fibrosis results from collagen deposition in the extracellular matrix and may be of both ischemic and non-ischemic origin, although it occurs more frequently following ischemia-induced cardiomyocyte injury [83].

CMR studies in runners suggest the presence of focal myocardial fibrosis in up to half of asymptomatic athletes. Contributing factors include training frequency, repeated microinjuries caused by prolonged exertion, pressure overload in the pulmonary artery, exercise-induced hypertension, and undiagnosed myocarditis [84,85]. In rats subjected to a 16-week running program—equivalent to approximately 10 years of endurance training in humans—eccentric myocardial hypertrophy, diastolic dysfunction, atrial enlargement, and collagen fiber deposition in the RV and both atria have been observed [86,87].

As a result of repeated episodes of microtrauma and insufficient recovery, progressive myocardial remodeling may occur. Additionally, excessively short intervals between intensive training sessions and inadequate recovery time promote the accumulation of reversible myocardial microinjuries [46].

Prolonged and intensive training, causing repetitive microtrauma, can lead to the loss of desmosomal integrity and structural damage to cardiomyocytes. These changes may result in myocardial fibrosis, particularly in the RV and atria [88,89]. Fibrosis of the myocardium—especially in the area of the right ventricular free wall and the interventricular septum—can lead to disturbances in cardiac mechanics and impaired function [90,91].

Some studies suggest that men may be more susceptible to developing fibrosis [84]. Moreover, increased secretion of pro-inflammatory cytokines (e.g., IL-6 and TNF-α) is associated with a higher risk of cardiac dysfunction [92,93,94].

Exercise-induced hypertension contributes to cardiomyocyte hypertrophy and expansion of the interstitial space due to fibrosis [95]. Elevated systolic blood pressure during exercise and longer running distances may promote the development of myocardial fibrosis. Additionally, individuals with hypertensive heart disease often present with non-ischemic myocardial fibrosis [96,97].

#### 2.2.2. Cardiac Hypertrophy

Physiological cardiac hypertrophy, an adaptive response to regular endurance-type physical activity, is associated with a range of beneficial structural and molecular processes. These include increased cardiomyocyte mass and regeneration, improved myocardial contractility, sarcomere reorganization, prolonged cell survival, favorable metabolic and mitochondrial adaptations, remodeling of the conduction system, and enhanced angiogenesis [98,99].

Secondary thickening of the interventricular septum may be related to the accumulation of type I and III collagen fibers and free collagen within the heart wall [100,101]. Endurance athletes often exhibit left ventricular chamber enlargement and mild eccentric hypertrophy, which enables an increase in cardiac output during exercise [29]. Physical activity may stimulate the activity of Akt kinase—a serine–threonine protein that plays a key role in cell proliferation and survival, as well as in adaptive and pathological myocardial remodeling processes. In animal models, it has been shown that after two weeks of intense exercise, Akt pathway expression in the heart induces reversible myocardial hypertrophy. However, after six weeks of strenuous training, irreversible cardiomyopathy develops, accompanied by reduced capillary density and progressive myocardial fibrosis. Studies involving patients with pathological cardiac hypertrophy and heart failure have shown elevated levels of angiotensin II (Ang II), endothelin-1 (ET-1), insulin-like growth factor 1 (IGF-1), and catecholamines compared to healthy individuals. ET-1 is a vasoconstrictive agent produced by endothelial cells and cardiomyocytes. Elevated plasma levels of ET-1 are associated with endothelial dysfunction, left ventricular hypertrophy, and diastolic dysfunction. On the other hand, regular aerobic exercise has been shown to reduce ET-1 levels.

IGF-1 promotes physiological cardiac hypertrophy via activation of the PI3K–Akt pathway. Ang II and ET-1 stimulate the development of pathological hypertrophy through activation of the mitogen-activated protein kinase (MAPK) and calcineurin pathways. These processes promote cardiomyocyte apoptosis and necrosis, replaced by excessive collagen deposition in the extracellular matrix. This leads to increased stiffness of the heart walls, systolic and diastolic dysfunction, and fibrosis of the conduction system, which may cause arrhythmias, including AF [34,49,102].

Endurance athletes, such as long-distance runners, are at increased risk of developing cardiac arrhythmias, including AF, ventricular tachycardia (VT), supraventricular tachycardia, and premature atrial and ventricular contractions [103]. A link has been shown between chronic, intense physical exertion and AF—the pathophysiological mechanisms include atrial stretch and enlargement, increased parasympathetic activity, atrial fibrosis, and recurrent episodes of myocarditis.

Atrial stretch and enlargement resulting from prolonged increased venous return and SV may promote the development of reentry—a mechanism leading to AF [104]. Atrial fibrosis and increased parasympathetic activity can modify the electrophysiological properties of the atria, shortening refractoriness and facilitating the initiation and maintenance of arrhythmias. Likewise, increased sympathetic activity and the related electrophysiological changes may contribute to the occurrence of ventricular arrhythmias in athletes [90,105].

#### 2.2.3. Vascular Alterations

Prolonged and intensive exercise may also negatively affect endothelial function. Studies have shown that after completing a marathon, there may be a transient impairment of endothelium-dependent vasodilation, indicating endothelial dysfunction. Mechanisms underlying this condition include elevated levels of free fatty acids, increased concentrations of pro-inflammatory cytokines (e.g., IL-6, TNF-α, E-selectin, ICAM-1, VCAM-1), and the presence of ROS, which can damage endothelial cells and disrupt the balance between pro- and anticoagulant factors [106,107].

Endothelial dysfunction may promote the development of atherosclerosis and thrombosis—these risks may be particularly relevant for individuals engaging in endurance sports under extreme conditions (high temperatures, dehydration, hypoxia) [107,108,109].

Arterial inflammation, triggered by elevated blood levels of free fatty acids, mechanical stress on vessel walls, and the presence of ROS, can lead to endothelial dysfunction and thrombus formation. Exercise-induced rhabdomyolysis promotes the release of von Willebrand factor from endothelial cells, and in combination with vasodilation and reduced plasma volume, may disrupt the balance between coagulation and fibrinolysis [110,111].

Persistently elevated CO in the early post-race period leads to increased wall tension and dilation of the right atrium and ventricle, resulting in the release of cytoplasmic markers—this effect is not necessarily related to cardiomyocyte necrosis [15,23,112].

Some data suggest that long-term, high-intensity exercise may lead to atherosclerotic changes in the coronary arteries—despite the generally favorable lipid profile of athletes. Vascular imaging in some marathon runners has revealed the presence of calcified atherosclerotic plaques, which may increase the risk of cardiovascular events, especially in the presence of additional risk factors such as hypertension, smoking, or genetic predisposition.

An increase in blood pressure during exercise—referred to as exercise-induced hypertension—may also contribute to myocardial hypertrophy and the development of fibrosis. Longer distances and higher training intensity increase this risk.

There is also evidence suggesting the possibility of subclinical myocardial infarction during very intense competitions. This may result from demand ischemia, microemboli, plaque rupture, or coronary artery spasm [39,40,113]. Elevated troponin levels may be observed in up to 10% of individuals who train regularly at high intensity [48].

Available data suggest that intense exercise may also lead to subclinical myocardial infarction caused by exercise-induced ischemia, microthrombi, plaque rupture, or coronary artery spasm [114].

Potential pathological changes include fibrosis at the right ventricular insertion point—observed in up to 40% of elite athletes—most likely related to mechanical stress and cardiomyocyte stretching. In less than 5% of athletes, subendocardial fibrosis has been reported, possibly associated with microthrombi or exercise-induced ischemia. In cases of more extensive fibrosis, involving both the subendocardial and intramyocardial layers, the likely cause is prior myocarditis or genetic disease [51,112,115].

#### 2.2.4. Left and Right Atrium Responses

Physiological changes observed in runners include increased pulmonary venous return, elevated preload, cardiac output, and LV filling [116]. These changes contribute to remodeling and enlargement of the LA. Intensive endurance training leads to volume overload and increased atrial pressure. Mechanisms promoting this process include parasympathetic dominance, enhanced neurohormonal activation (e.g., increased secretion of catecholamines and natriuretic peptides), and recurrent afterload elevations induced by training—all of which support LA remodeling [117]. In young women, higher estrogen levels improve myocardial compliance and elasticity, enhance vascular function, and promote better overall cardiac performance [118,119,120,121,122,123,124].

The size of the RA adapts to the increased preload of the RV, allowing for greater blood volume intake and increased cardiac output [125]. Studies have documented exercise-induced right ventricular cardiomyopathy, attributed to increased wall stress, which may lead to cell injury and the release of cardiac biomarkers immediately post-exercise. In runners, late gadolinium enhancement has been observed at RV insertion points on CMR, likely resulting from pulmonary artery overload and repetitive microtrauma during intense physical activity [89,126].

#### 2.2.5. Ventricular Responses

Physiological left ventricular hypertrophy—an adaptive response to regular moderate or intense exercise—is associated with beneficial processes such as cardiomyocyte growth and regeneration, enhanced contractility, sarcomere remodeling, improved cell survival, metabolic and mitochondrial adaptations, conduction system remodeling, and increased angiogenesis [98,99]. Accumulation of type I and III collagen fibers and free collagen in the interventricular septum contributes to its secondary thickening [100,101]. Chronic volume overload may explain the elevated left ventricular end-diastolic volume index (LVEDVi) [28]. Endurance athletes often show increased LV internal diameter and mild eccentric hypertrophy, enabling sufficient cardiac output during exercise [29]. The duration of diastole depends on heart rate, while LV filling is influenced by hydration status and blood flow redistribution; blood pressure affects afterload [127]. Prolonged volume overload and excessive sympathetic activation may lead to the development of heart failure with preserved ejection fraction (HFpEF), and less commonly, heart failure with reduced ejection fraction (HFrEF). Diagnosing such conditions in athletes can be particularly challenging, as standard echocardiographic parameters (e.g., ejection fraction) often remain within normal limits [90]. The presence of fibrosis reduces ventricular diastolic compliance, contributing to atrial enlargement, the development of AF and HFpEF [128].

**Figure 2:**
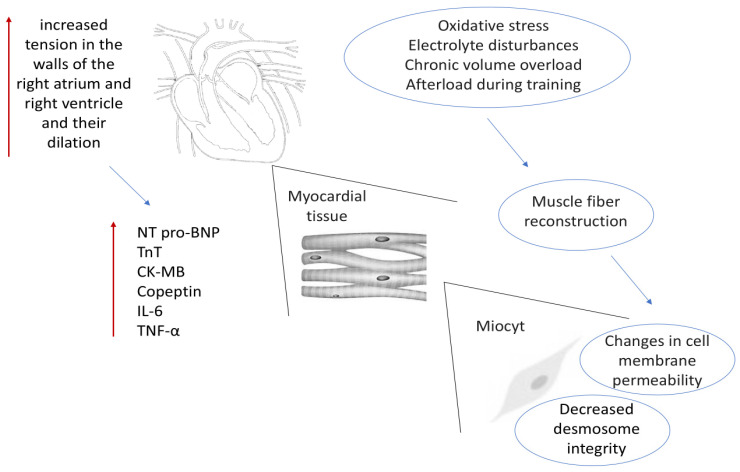
Diagram of molecular changes during and after exercise.

During a marathon, increased pulmonary artery pressure leads to elevated end-systolic wall stress in the RV. As exercise intensity and duration increase, impaired RV contractility may explain the elevated right ventricular end-systolic volume index (RVESVi). Mild reduction in RV systolic function in runners is considered a manifestation of increased contractile reserve [23,30,31,32]. During intense endurance activity, cardiac output may increase up to sevenfold compared to resting values, leading to increased pulmonary circulation pressure and heightened RV wall stress [23,129] (Figure 2).

## 3. Detection Parameters

### 3.1. Echocardiography

Electrocardiographic changes in endurance athletes are primarily the result of increased vagal tone and relative cardiac enlargement. The most commonly observed findings include sinus bradycardia, first-degree atrioventricular (AV) block and Mobitz type I block, increased QRS complex voltage, incomplete right bundle branch block, and early repolarization [15,110]. The first reports describing electrocardiographic abnormalities in athletes—correlating with myocardial hypertrophy and training status—were published in the 1970s [130]. In 1980, abnormalities in repolarization were documented in endurance athletes. These included pseudo-ischemic T-wave inversions at rest, which normalized with maximal exercise or isoprenaline administration. In some individuals with T-wave inversion, asymptomatic mitral valve prolapse and echocardiographic patterns of asymmetric ventricular septal hypertrophy were also observed [131,132].

In general, ECG findings in endurance athletes reflect physiological adaptations to regular, intense training, collectively known as “athlete’s heart.” When asymptomatic, these findings do not require further evaluation. To aid in distinguishing benign from pathological changes, the “Seattle Criteria” were developed. A resting ECG in an athlete may include the following:

- sinus bradycardia (≥30 bpm),
- sinus arrhythmia,
- ectopic atrial rhythm,
- junctional escape rhythm,
- first-degree AV block (PR interval > 200 ms),
- Mobitz type I second-degree AV block (Wenckebach),
- incomplete right bundle branch block (nRBBB),
- voltage criteria for left ventricular hypertrophy (LVH),
- early repolarization (ST-segment or J-point elevation, J-waves, or terminal QRS slurring),
- convex ST-segment elevation with T-wave inversion in leads V1–V4 (particularly in Black athletes) [133].

In contrast, abnormal ECG findings in athletes that warrant further investigation include the following:

- T-wave inversion,
- ST-segment depression,
- pathological Q-waves,
- complete left bundle branch block (LBBB),
- intraventricular conduction delay,
- left axis deviation (−30° to −90°),
- signs of left atrial enlargement (P-wave > 120 ms in leads I and II),
- right ventricular hypertrophy,
- frequent premature ventricular complexes (PVCs),
- prolonged or shortened QT interval,
- Brugada-like ECG pattern,
- profound sinus bradycardia (<30 bpm),
- atrial tachyarrhythmias,
- ventricular arrhythmias [133,134,135].

Recognizing and correctly interpreting these findings is critical for the prevention of sudden cardiac death (SCD), regardless of whether the ECG is performed as part of a diagnostic or screening evaluation. Recent studies on athlete ECGs focus predominantly on professional athletes, particularly football players, while data on amateur athletes, such as marathon runners, remain limited.

The most commonly observed resting ECG abnormalities in amateur marathon runners include sinus bradycardia, first-degree AV block (AVB I°), incomplete right bundle branch block (nRBBB), voltage criteria for LVH, early repolarization (ER), and left axis deviation (LAD). Both bradycardia and AVB tend to normalize in ECGs taken after a marathon. At that time, signs of right atrial enlargement (RAE) and left atrial enlargement (LAE) become more prevalent, likely due to adrenergic stimulation. The prevalence of LVH and ER remains consistent in both pre- and post-race recordings.

Isolated ECG parameters that tend to change post-race include an elevated heart rate, increased P-wave amplitude (notably in men), and a shortened QT interval, including QT, QTc, QTmin, and QTmax durations [136,137].

In summary, endurance athletes, including amateurs, commonly exhibit ECG changes that reflect physiological cardiac adaptation to chronic physical exertion. Among the most frequently observed are sinus bradycardia, considered an early electrophysiological adaptation, and first-degree AV block, typically appearing at a later stage and possibly associated with progressive structural myocardial remodeling.

This adaptation process progresses gradually. Initial ECG changes can occur with moderate physical activity (>3 h/week), while the characteristic ECG profile of elite athletes usually develops after several years of training, once cumulative exercise time exceeds 3000 h.

Although most ECG abnormalities fall within the spectrum of physiological adaptation and require no further assessment, approximately 15% of findings may indicate clinically significant conditions unrelated to training. Moreover, male athletes appear to be more prone to exercise-induced abnormalities such as QTc prolongation and right atrial overload, potentially increasing their risk of post-exercise arrhythmias and sudden cardiac death.

ECG interpretation should consider individual characteristics such as age, sex, and race—factors particularly relevant when assessing adolescent athletes, whose cardiac remodeling is dynamic both before and during puberty.

Given the high prevalence of both adaptive and potentially pathological changes, even among amateur athletes, routine ECG screening appears justified regardless of training volume [136,137,138,139].

Based on available evidence, cardiovascular assessment of endurance athletes should not rely solely on resting ECG, which lacks sufficient sensitivity for detecting exercise-induced arrhythmias. When initial abnormalities, clinical symptoms, or suspected concealed pathology are present, 24 h Holter monitoring is a valuable diagnostic tool. It enables assessment of cardiac rhythm during real-life activities, allowing for detection of arrhythmias that may not be evident on a resting ECG.

Holter monitoring is especially recommended in individuals with ≥2 PVCs on ECG or a daily PVC burden exceeding 2000, as these parameters are associated with a higher risk of cardiomyopathy. A high number of ectopic beats, non-sustained VT, or worsening of arrhythmias during exercise are key indications for further evaluation, including cardiac magnetic resonance imaging and, in selected cases, electrophysiological studies.

Holter monitoring also facilitates the assessment of repolarization variability—particularly in the context of abnormal T-wave inversion—which may signal concealed cardiomyopathy. In athletes with a positive family history of inherited cardiac conditions or those with exercise-induced syncope or palpitations, ambulatory rhythm monitoring is a crucial component of comprehensive diagnostic evaluation, enabling arrhythmia detection beyond the clinical setting.

In conclusion, although the 12-lead ECG remains the first-line screening tool, its interpretation must be contextualized. Dynamic and individualized rhythm assessment methods, such as Holter monitoring, are essential for thorough cardiovascular evaluation [135,140,141,142,143].

### 3.2. Transthoracic Echocardiography

Transthoracic echocardiography (TTE) remains the primary imaging modality for evaluating cardiac structure and function due to its wide availability, non-invasiveness, repeatability, and low cost. For these reasons, it is also frequently employed in the assessment of athletes.

#### 3.2.1. Assessment of Left and Right Atrium

In both professional and recreational athletes with long-term exposure to endurance training, enlargement of the LA has been observed in comparison to sedentary individuals [144,145,146,147,148,149,150]. Immediately following exercise, a transient decrease in LA size may occur, which can be attributed, among other factors, to dehydration [116]. Unlike in the general population, where LA enlargement is typically associated with elevated LV filling pressures and diastolic dysfunction, in athletes, LA enlargement is often a physiological adaptation to chronic volume overload, adrenergic stimulation, and increased LV end-diastolic volume (EDV), and does not necessarily indicate pathology [151,152]. A reduction in global LA strain has also been reported [148,150,153]. Although increased LA volume correlates with a higher incidence of AF, interpreting this relationship in athletes remains challenging.

Data on the effects of endurance training on the right RA are more limited. Current evidence suggests that endurance training does not significantly affect RA morphology or function in amateur runners [116]. However, in long-term endurance athletes, RA volume enlargement and decreased RA strain have been observed [144,154].

#### 3.2.2. Assessment of Left and Right Ventricle

In individuals engaged in regular endurance training, an approximately 15% increase in LV wall thickness and a 10% increase in volume have been reported. These changes vary by sex, age, and ethnicity and are typically considered physiological adaptations [155,156,157,158]. Assessment of LV wall thickness plays a key role in differentiating physiological from pathological hypertrophy, such as hypertrophic cardiomyopathy (HCM). Wall thickness values in the range of 13–14 mm are considered to fall into the so-called “gray zone,” whereas asymmetric hypertrophy or thickness ≥15 mm may indicate underlying structural pathology [159]. In response to volume overload, increases in both LV and LA volumes are observed. Among endurance athletes, including recreational marathon runners, adaptive cardiac remodeling features such as chamber enlargement (especially of the LA, but also increased LV end-diastolic and end-systolic diameters) are commonly observed [46,158,160,161,162,163]. An increase in LV mass index is also frequently reported [160,161,162,163,164]. Interestingly, one study found that training-related chamber enlargement without myocardial hypertrophy was more common in women, whereas in men, LVH often accompanied volume expansion [163]. The increase in LV size is often associated with increased LA volume, which may predispose individuals to supraventricular arrhythmias [165]. LVEF in endurance athletes is typically normal or slightly reduced at rest [160,166,167]. This reduction may be explained by the elevated end-diastolic volume—an enlarged ventricle requires less contractile force to maintain adequate cardiac output.

Differences in LV global longitudinal strain (GLS) between athletes and healthy controls are generally not significant [157,164,167,168,169], but a reduced GLS may indicate subclinical systolic dysfunction in physically active individuals. A transient post-exercise decrease in LV GLS has also been documented, likely reflecting myocardial adaptation to significant hemodynamic load [155,156].

Among trained individuals, the E/A ratio is typically normal or mildly elevated [155,157,158]. Higher E/A ratios compared to the general population often result from reduced A-wave velocity, suggesting a lower contribution of atrial contraction to LV filling due to increased preload [144]. In endurance athletes, the E/e’ ratio generally remains within normal limits [165]. Since diastolic duration is heart rate-dependent, LV preload can fluctuate due to dehydration and blood flow redistribution, while blood pressure influences afterload. Thus, reductions in E/A and e’ following exercise may not reflect true impairment of LV filling, particularly in the absence of corresponding changes in E/e’ [127].

Endurance training subjects the RV to chronic pressure and volume overload, resulting in RV volume enlargement [166]. As with the LV, this may be accompanied by a slight reduction in RV ejection fraction (RVEF), which usually remains within the normal range [170].

Studies assessing the impact of endurance exercise on RV systolic function have demonstrated lower values of RV free wall longitudinal strain (FWLS) and basal segment strain compared to control subjects. However, global RV systolic indices—such as RV GLS and tricuspid annular plane systolic excursion (TAPSE)—typically remain unchanged, although intense exercise may lead to transient reductions in RV function [168,171]. In elite endurance athletes, resting RV GLS reduction is interpreted as a manifestation of physiological remodeling rather than myocardial injury [125].

In addition to reduced basal segment strain, athletes often exhibit increased apical RV strain contribution, resulting in a more pronounced base-to-apex strain gradient. This pattern may represent an adaptation to chronic pressure and volume overload [172].

A study in recreational marathon runners demonstrated increased RV EDV and enhanced RV systolic function-assessed using parameters such as RVEF, 3D RV fractional area change (FAC), 3D TAPSE, and longitudinal strain. These values were higher in marathon runners compared to sedentary individuals, suggesting RV adaptation to regular physical activity [93]. Interestingly, other studies have reported lower RVEF values in endurance athletes compared to the general population [166,170].

RV FWLSR measured during stress echocardiography may help distinguish physiological adaptations from pathological findings related to arrhythmogenic right ventricular cardiomyopathy (ARVC). Special attention should be paid to a lack of increase—or abnormally low FWLSR values—during exercise, as these may indicate subclinical RV dysfunction or the presence of arrhythmias [173].

The current evidence suggests that RV strain assessment offers valuable insights into RV adaptive capacity and may serve as a useful tool for monitoring right ventricular function.

## 4. Conclusions

Regular cardiologic examinations, including echocardiography, resting and exercise ECG, and monitoring of cardiac biomarkers, are essential for detecting pathological changes. Advanced imaging methods such as CMR can aid in identifying myocardial fibrosis, which in some athletes may increase the risk of arrhythmias and heart failure. The long-term consequences of intensive training may include an increased risk of cardiac arrhythmias, and in rare cases, the development of cardiomyopathy.

Although physical activity has well-documented health benefits, caution is warranted, especially in athletes with a family history of cardiovascular disease or prior cardiac issues. To minimize the risk of complications, regular screening is recommended—particularly for competitive athletes.

Importantly, the presence of comorbidities such as diabetes, hypertension, and heart failure with preserved ejection fraction (HFpEF) may substantially influence both exercise tolerance and cardiac responses to training. Diabetes and hypertension accelerate vascular stiffening, impair endothelial function, and increase the propensity for maladaptive hypertrophy and fibrosis, thereby amplifying the hemodynamic stress of endurance exercise. Likewise, athletes with HFpEF may demonstrate reduced diastolic compliance, impaired ventricular filling, and a greater likelihood of exercise-induced elevations in filling pressures, which can limit performance and predispose to atrial arrhythmias. These comorbid conditions may attenuate the beneficial adaptations typically observed in the athlete’s heart, shifting the balance toward pathological remodeling. Accordingly, careful risk stratification and individualized exercise prescriptions are essential when managing athletes with such comorbidities.

Individualized training plans that incorporate adequate recovery periods, along with close monitoring for symptoms such as dyspnea, palpitations, or sudden drops in performance, are critical. Education on potential risks and early preventive strategies can help avoid serious health outcomes.

## Figures and Tables

**Table 1:** Biomarkers associated with intense physical exercise and their clinical significance.

Biomarker	Type/Role	Post-Exercise Changes	Clinical Significance	Mechanism/Notes
Cardiac Troponins T/I (cTnT, cTnI)	Cardiomyocyte injury	Increase (often transient)	Reversible myocardial injury, may mimic ACS	Increased sarcolemmal permeability, mechanical stress, reactive oxygen species (ROS)
CK-MB	Myocardial injury	Increase, especially in RV	Classical marker of necrosis; elevated in ~8% of athletes	Released from damaged cardiomyocytes
NT-proBNP	Cardiac wall stress	5–10× increase	Indicates volume overload, neurohormonal adaptation	Correlates with duration and intensity of exercise
MR-proADM	Hemodynamic stress	Increase	Reflects hemodynamic overload	Indirectly associated with endothelial function
sST2	IL-33 receptor/mechanical strain	Increase (notably in males)	Marker of mechanical stress and overload	IL-33/ST2 axis activation; fibrosis inhibition
Galectin-3 (Gal-3)	Fibrosis/cardiac remodeling	Increase, especially with reduced LVEF	Marker of myocardial fibrosis and remodeling	Secreted by macrophages; correlates with RV function and LVEF
TIMP-1, CITP, PICP	Collagen turnover	Increase	Reflect myocardial remodeling and fibrosis	Indicators of collagen synthesis and degradation
sVCAM-1	Inflammation/LA remodeling	Increase	Correlates with LA volume; reflects atrial remodeling	Involved in leukocyte adhesion
IL-6, TNF-α, IL-8, IL-10	Pro- and anti-inflammatory cytokines	Increase (variable dynamics)	Inflammatory response, tissue regeneration	IL-6 promotes M2 activation and supports regeneration
Copeptin	Endogenous stress	Increase	Early marker of stress and dehydration	Correlates with AVP; used in acute syndrome diagnostics
GDF-15	Metabolic/mitochondrial stress	Increase	Marker of systemic stress and adaptation	Induced by cellular stress
FGF-21	Muscle metabolism/mitochondria	Increase	Involved in mitophagy and muscle fiber regulation	Activates PI3K-Akt, ATF4 pathways
BDNF	Cardiomyocyte proliferation/protection	Increase	Supports cardiomyocyte survival and angiogenesis	Acts via TrkB receptor
Follistatin	Myogenesis/myostatin inhibition	Increase	Promotes protein synthesis and muscle regeneration	Inhibits SMAD3; activates mTOR/S6K signaling
Myostatin	Inhibits muscle growth	Decrease	Reduces muscle mass and strength	Activates SMAD2/3; promotes muscle catabolism
Musclin	Muscle adaptation/oxidation	Decrease	May support mitochondrial biogenesis	Correlates with IL-10 and IL-8
LDH	Tissue injury	Increase	Correlates with cytokines, not with myokines	Indicates cellular breakdown

ACS, acute coronary syndrome, ATF4, activating transcription factor 4, AVP, arginine vasopressin, BDNF, brain-derived neurotrophic factor, CK-MB, creatine kinase myocardial band, cTnI, cardiac troponin I, cTnT, cardiac troponin T, CITP, carboxy-terminal telopeptide of type I collagen, FGF-21, fibroblast growth factor 21, Gal-3, galectin-3, GDF-15, growth differentiation factor 15, IL-6, interleukin-6, IL-8, interleukin-8, IL-10, interleukin-10, IL-33, interleukin-33, LA, left atrium, LDH, lactate dehydrogenase, LVEF, left ventricular ejection fraction, MR-proADM, mid-regional pro-adrenomedullin, mTOR, mechanistic target of rapamycin, NT-proBNP, N-terminal pro–B-type natriuretic peptide, PICP, procollagen type I C-terminal propeptide, PI3K, phosphoinositide 3-kinase, RV, right ventricle, ROS, reactive oxygen species, sST2, soluble suppression of tumorigenicity 2, sVCAM-1, soluble vascular cell adhesion molecule-1, SMAD2/3, mothers against decapentaplegic homolog 2/3, TIMP-1, tissue inhibitor of metalloproteinases-1, TNF-α, tumor necrosis factor alpha, TrkB, tropomyosin receptor kinase B. Own elaboration.

## Data Availability

Data are contained within the article.
